# Cytotoxic and Antifungal Activities of Diverse α-Naphthylamine Derivatives

**DOI:** 10.3797/scipharm.1209-03

**Published:** 2012-10-23

**Authors:** Vladímir V. Kouznetsov, Susana A. Zacchino, Maximiliano Sortino, Leonor Y. Vargas Méndez, Mahabir P. Gupta

**Affiliations:** 1 Laboratorio de Química Orgánica y Biomolecular, Escuela de Química, Universidad Industrial de Santander, A. A. 678, Bucaramanga, Colombia.; 2 Farmacognosia, Facultad de Ciencias Bioquímicas y Farmacéuticas, Universidad Nacional de Rosario, Suipacha 531, (2000) Rosario, Argentina.; 3 Grupo de Investigaciones Ambientales, Facultad de Química Ambiental, Universidad Santo Tomás, A. A. 1076, Bucaramanga, Colombia.; 4 Centro de Investigaciones Farmacognósticas de la Flora Panameña (CIFLORPAN), Facultad de Farmacia, Universidad de Panamá, Panamá, Republic of Panama.

**Keywords:** α-Naphthylamines, Antifungal properties, Cytotoxic agents

## Abstract

Diverse α-naphthylamine derivatives were easily prepared from corresponding aldimines derived from commercially available α-naphthaldehyde and anilines or isomeric pyridinecarboxyaldehydes and α-naphthylamine. The secondary amines obtained were tested as possible antifungal and cytotoxic agents. The diverse *N*-aryl-*N*-[1-(1-naphthyl)but-3-enyl]amines obtained were active (IC_50_ < 10 μg/mL) against breast (MCF-7), non-small cell lung (H-460), and central nervous system (SF-268) human cancer cell lines, while *N*-(pyridinylmethyl)-naphthalen-1-amines resulted in activity against (MIC 25–32 μg/mL) some human opportunistic pathogenic fungi including yeasts, hialohyphomycetes, and dermatophytes.

## Introduction

Secondary amines that include aromatic planar rings (e.g. aryl, naphthyl, etc.) are widely used throughout the chemical industry as basic intermediates to prepare pharmaceuticals, agrochemicals, and fine chemicals [1–5]. Moreover, they are one of the most common structural features of naturally occurring, biologically active compounds [6]. Due to their unique biological properties, these compounds have played an important role in chemo-therapeutic approaches in a variety of diseases, including antiparasitic infectious diseases [2]. However, it is known that naphthylamines could be carcinogenic for humans [7–9]. These compounds have still demonstrated interesting pharmacological models in biomedical studies [10–12]. As a result, the chemistry and biology of these compounds have received considerable attention from both the theoretical and practical points of view.

**Fig. 1.**
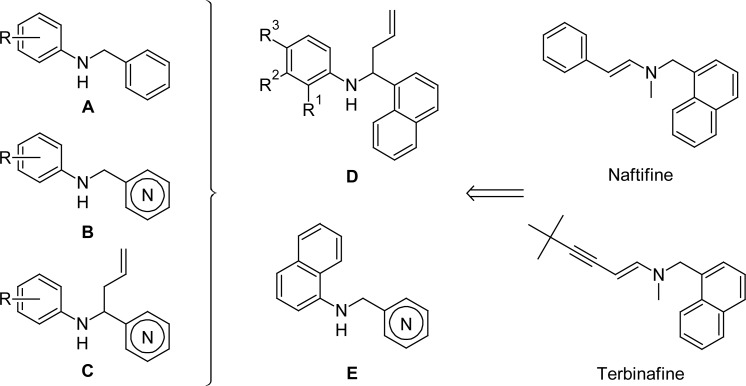
Structural similitude of allylamine drugs and studied α-naphthylamines

As a part of our drug discovery program, we have been actively involved in determining the features that are important for antifungal activities of different amines and we found that *N*-benzyl anilines (type **A**), or *N*-pyridinylmethylanilines (type **B**), and *N*-aryl-*N*-(1-pyridinylbut-3-enyl)amines (“homoallylamines”) (type **C**) (Figure 1), easily accessible from aldimines, displayed significant activity against some pathogenic dermatophytes [13–18].

These facts encouraged us to develop other naphthylamine derivatives and evaluate them for their antifungal activity and in parallel their cytotoxic properties. So, we report herein the biological results of a new series of *N*-aryl-*N*-[1-(1-naphthyl)but-3-enyl]amines (type **D**) and *N*-(pyridinylmethyl)naphthalen-1-amines (type **E**) that could be considered as structural analogues of the antifungal drugs naftifine and terbinafine, called “allylamine derivatives” [19] (Fig. 1).

## Results and Discussion

### Chemistry

The desired naphthylamine derivatives **5** and **6** were prepared from commercially available aromatic aldehydes α-naphthaldehyde **1** and isomeric pyridinecarboxyaldehydes **2a–c** and anilines **3** or α-naphthylamine **4** in easy two-step synthesis protocols via initial *N*-aryl aldimines formation.

These aldimines, either by nucleophilic addition of allylmagnesium bromide to the C=N bond or the reduction with an excess of NaBH_4_ in methanol, produced the corresponding secondary and *N*-aryl-*N*-[1-(1-naphthyl)but-3-enyl]amines **5a–f**[18] (“homoallylamines”, type **D**) (route a, Scheme 1) and *N*-(pyridinylmethyl)naphthalen-1-amines **6a-c** (type **E)** (route b, Scheme 1), respectively.

These secondary amines were obtained as colored solids or viscous oils in 80–95% yields after purification using a SiO_2_ chromatography column (Table 1) and were strongly characterized by the spectral data.

### Biological properties

These two series of compounds were tested for cytotoxic activities against breast (MCF-7), non-small cell lung (H-460), and central nervous system (SF-268) human cancer cell lines [20, 21] and for antifungal properties against a panel of 10 human opportunistic pathogenic fungi including yeasts, *Aspergillus* spp. and dermatophytes [22, 23] (Table 2).

Results showed (Table 2) that the tested compounds of type **D**, and all *N*-aryl-*N*-[1-(naphth-1-yl)but-3-enyl]amines **5a–e,** but not **5f,** showed cytotoxicity with IC_50_ ≤ 10 μg/mL, making them potential anticancer agents. Of the three tested cell lines, MCF-7 and H-460 were the most susceptible ones. From these results, certain conclusions relating to the structure with the cytotoxic activity can be drawn: (i) the donor substituent OMe as R^3^ renders the most cytotoxic compound from both points of view with the lower IC_50_ value and the broader spectrum of action [compare activities of **5a** (R^3^ = MeO); **5b** (R^3^ = F); **5c** (R^3^ = Cl); **5d** (R^3^ = Br)]; (ii) the comparison of the activity of compounds possessing a halogen as R^3^ showed that a Br (**5b**, **5c** and **5d**) confers better activity than a chloro (Cl) atom, followed by a fluorine (F) atom; (iii) the addition of an extra Cl as R_2_ to **5c** leads to the equipotent **5e**, suggesting that a halogen in the *meta* position does not modify the activity; (iv) in contrast, the addition of an extra F as R^1^ to **5b** leads to **5f**, which shows a loss of activity in the three cell lines. This result could be related to the steric hindrance resulting of a substituent on the *ortho* position.

Regarding the results obtained from compounds type **B**, it appears that the position of the N atom in the pyridine ring of *N*-(pyridinylmethyl)naphthalen-1-amines **6a–c** exert some influence in the activity since compounds possessing α- or β-pyridine rings (but not that γ-pyridine moiety) are inactive.

Results of the antifungal assays of type **D** molecules showed that the cytotoxic compounds **5a–e** were completely devoid of antifungal properties, clearly suggesting a selective toxicity of these compounds against cancer cell lines.

For a better understanding of the antifungal results of compounds **6a**–**c**, their cytotoxic and antifungal behaviors are shown in Table 3, in which the antifungal results of **6a–c** in each fungal species is included.

Results showed that among compounds **6**, molecule **6b** displayed moderate antifungal activity with a broad spectrum of action, with *T. rubrum* being the most susceptible species (MIC = 6.25 μg/mL). In addition, it did not show cytoxicity at the concentration at which it was antifungal. Compound **6c** showed moderate antifungal activity (MIC = 32–62 μg/mL) only against dermatophytes. However, at much lower concentrations (IC_50_ = 3.3–4.6 μg/mL) it was cytotoxic against the three tested cell lines.

Taking into account all of these results, we can say that among molecules **6**, compounds **6b** and **6c** can be optimized in forthcoming works, considering that compound **6c** is the best cytotoxic candidate and **6b** the best antifungal against *T. rubrum*. This result is very interesting since this fungal species is responsible for approximately 80–93% of chronic and recurrent dermatophyte infections in human beings. They are the etiological agent of tinea unguium (producer of invasive nail infections), tinea manuum (palmar and interdigital areas of the hand infections), and tinea pedis (Athlete’s foot), the last one being the most prevalent fungal infection in developed countries, and the first one accounting for 50% and 90% of all fingernail and toenail infections, respectively, and new agents with high activity against *T. rubrum* are highly welcomed.

In summary, we have analyzed the cytotoxic and antifungal properties of some α-naphthylamine derivatives prepared from available aldimines. *N*-Aryl-*N*-[1-(naphth-1-yl)but-3-enyl]amines **5a–e** showed good selective cytotoxic activities against three cancer cell lines being the one containing an OMe as the most active substituent (**5a**). In turn, among the three *N*-(pyridinylmethyl)naphthalen-1-amines **6a–c**, the one possessing the pyridine ring with N in the β-position (comp. **6b**) showed moderate activity mainly against *T. rubrum*, while it is not cytotoxic at the same concentrations.

## Experimental

### Chemistry

IR spectra were recorded on a Lumex Infralum FT-02 spectrophotometer. ^1^H and ^13^C NMR spectra were measured on a Bruker AM-400 spectrometer (400 MHz ^1^H NMR and 100 MHz ^13^C NMR), using CDCl_3_ as the solvent. TMS was used as an internal standard. Chemical shifts (δ) and *J* values are reported in ppm and Hz, respectively. A Hewlett Packard 5890a Series II Gas Chromatograph interfaced to an HP 5972 Mass Selective Detector with an HP MS ChemStation Data system was used for MS identification at 70 eV using a 60 m capillary column coated with HP-5 [5% phenylpoly(dimethylsiloxane)]. Melting points were measured on a Fisher Johns melting point apparatus. The reaction progress was monitored using thin layer chromatography on Silufol UV 254 TLC aluminum sheets. Column chromatography was carried out using silica gel (230–400 mesh). All reagents were purchased from Sigma and Aldrich Chemical Co. and used without further purification.

Synthesis of the secondary amines **5** and **6** was performed according to literature reports [17, 18]. Spectral data for known amines **5** were identical to those published in our work [18].

### Spectral Data for unknown amines 6

Comp. **6a**[24]: dark red viscous liquid. IR (neat), *v* (cm^−1^): 3386, 3054, 2838, 1589, 1527, 771; EM (IE), m/z (%): 234 (M+., 100), 204 (5), 156 (40), 142 (4), 128 (20), 115 (28). ^1^H NMR (400 MHz, CDCl_3_, Me_4_Si) δ 8.57 (dd, J = 7.5, 1.6 Hz, 1H), 8.09–8.02 (m, 2H), 7.72 (m, 1H), 7.60–7.51 (m, 3H), 7.39–7.30 (m, 3H), 6.97 (d, J = 7.8, 1.4 Hz, 1H), 4.70 (s, 2H), 3.98 (br s, 1H); ^13^C NMR (100 MHz, CDCl_3_, Me_4_Si), δ (ppm): 157.8, 148.8, 142.9, 136.4, 134.1, 128.4, 126.5, 125.6, 124.5, 123.3, 121.9, 121.4, 120.2, 117.2, 104.5, 48.8. Anal. Calcd. for C_16_H_14_N_2_: C, 82.02; H, 6.02; N, 11.96. Found: C, 81.96; H, 6.13; N, 11.53.

Comp. **6b**: red oil, IR (neat), *v* (cm^−1^): 3355, 3039, 1581, 1527, 771; EM (IE); m/z (%): 234 (M^+^, 100), 204 (3), 142 (63), 115 (88), 92 (19), 65 (17). ^1^H NMR (400 MHz, CDCl_3_ Me_4_Si), δ (ppm): 8.65 (s, 1H), 8.50 (d, *J* = 7.5 Hz, 2H), 8.08–8.01 (m, 2H), 7.88 (dd, *J* = 7.5, 1.4 Hz, 1H), 7.58–7.51 (m, 3H), 7.37 (each d, *J* = 7.5 Hz, 2H), 6.98 (dd, *J* = 7.5, 1.6 Hz, 1H), 4.36 (s, 2H), 4.02 (br s, 1H); ^13^C NMR (100 MHz, CDCl_3_ Me_4_Si), δ (ppm): 156.7, 148.6, 142,4, 136.4, 134.3, 128.8, 126.5 (2C), 125.9, 123.3, 122.2 (2C), 119.9, 118.2, 104.8, 48.1. Calcd. for C_16_H_14_N_2_: C, 82.02; H, 6.02; N, 11.96. Found: C, 81.87; H, 6.16; N 12.01.

Comp. **6c**: yellow oil, IR (neat), *v* (cm^−1^): 3299, 3050, 2881, 1589, 1543, 767; EM (IE), m/z (%): 234 (M^+.^, 100), 206 (2), 156 (13), 142 (45), 128 (18), 115 (67), 65 (7). ^1^H NMR (400 MHz, CDCl_3_, Me_4_Si) δ (ppm): 8.58 (each dd, *J* = 7.6, 1.4 Hz, 2H), 8.06–8.01 (m, 2H), 7.55–7.51 (m, 3H), 7.35–7.38 (m, 3H), 6.96 (dd, *J* = 7.5, 1.4 Hz, 1H), 4.35 (s, 2H), 4.00 (br s, 1H); ^13^C NMR (101 MHz, CDCl_3_, Me_4_Si) δ (ppm): 150.0 (2C), 148.7, 142.5, 134.4, 128.8, 126.5, 126.0, 125.0, 123.4, 122.2 (2C), 119.9, 118.1, 105.0, 47.2. Calcd. for C_16_H_14_N_2_: C, 82.02; H, 6.02; N, 11.96. Found: C, 81.78; H, 6.19; N, 11.85.

### Bioassays

#### Cytotoxic susceptibility testing

The cytotoxic activity was determined according to the method of Monks et al. Briefly, the three human cells lines [breast (MCF-7), non-small cell lung (H-460), and central nervous system (SF-268), obtained from the U.S. National Cancer Institute] were counted, diluted with fresh medium, and added to 96-well microtiter plates(100 μL/well) containing test materials (1 mg in 100 μL in DMSO). Test plates were incubated for 2 days at 37 °C in a 5% CO_2_ incubator. All treatments were performed in duplicate. After the incubation periods, cells were fixed by addition of 50 μL of cold 50% aqueous TCA solution (4°C for 60 min.), washed 4–5 times with tap water, and air-dried. The fixed cells were stained with 100 μL sulforhodamine B (SRB) (0.4% wt/vol. in 1% acetic acid) for 15 min. Free SRB solution was then removed by rinsing with 1% acetic acid (× 5). The plates were then air-dried, the bound dye was solubilized with 100 μL of 10 mM tris-base, and absorbance was determined at 515 nm using an ELISA plate reader (Bio-Tek Instruments, Inc. Model ELX-800). Finally, the absorbance values obtained with each of the treatment procedures were averaged, and the averaged value obtained with the zero day control was subtracted, measuring in this way the relative cell growth or unviability in treated and untreated cells. From the curves, growth inhibition (or growth stimulation) and 50% inhibition of growth (IC_50_) was calculated [21,22]. Adriamycin was used as the reference compound. The procedure for cell viability measurement was evaluated by a colorimetric method with resazurin. The macrophages J774 were seeded (5 × 10^4^ cells/well) in 96-well flat-bottom microplates with 100 μL of RPMI 1640 medium. The cells were allowed to attach for 24 h at 37 °C, 5% CO_2_ and the medium was replaced by different concentrations of the drugs in 200 μL of medium, and exposed for another 24 h. Growth controls were also included. Afterwards, a volume of 20 μL of the 2mM resazurin solution was added and plates were returned to the incubator for another 3 h to evaluate cell viability. The reduction of resazurin was determined by dual wavelength absorbance measurement at 490 nm and 595 nm. The background was subtracted. Each concentration was assayed in triplicate. The medium and drug controls were used as blanks in each test. Compounds with IC_50_ ≥ 10 μg/mL were considered inactive (not cytotoxic).

#### Antifungal activity

For the antifungal evaluation, standardized strains from the American Type Culture Collection (ATCC), Rockville, MD, USA, and CEREMIC (CCC), Centro de Referencia en Micología, Facultad de Ciencias Bioquímicas y Farmacéuticas, Suipacha 531-(2000)-Rosario, Argentina, were used: *C. albicans* ATCC 10231, *C. tropicalis* CCC 191, *S. cerevisiae* ATCC 9763, *C. neoformans* ATCC 32264, *A. flavus* ATCC 9170, *A. fumigatus* ATTC 26934, *A. niger* ATCC 9029, *T. rubrum* CCC 110, *T. mentagrophytes* ATCC 9972 and *M. gypseum* CCC 115. Strains were grown on Sabouraud-chloramphenicol agar slants for 48 h at 30 °C, maintained on slopes of Sabouraud-dextrose agar (SDA, Oxoid) and sub-cultured every 15 days to prevent pleomorphic transformations. Inocula of spore suspensions were obtained according to reported procedures [22, 23] and adjusted to 1–5 ×10^3^ spores with colony forming units (CFU)/mL.

#### Antifungal susceptibility testing

Broth microdilution techniques were performed following the guidelines of the CLSI for yeasts [22] and for filamentous fungi [23]. MIC values were determined in RPMI-1640 (Sigma) buffered to pH 7.0 with MOPS (Sigma). Microtiter trays were incubated at 35 °C for yeasts and hyalohyphomycetes and at 28 °C for dermatophyte strains in a moist, dark chamber; MICs were recorded at 48 h for yeasts, and at a time according to the amount of control fungus growth, for the rest of fungi. The susceptibilities of the standard drugs ketoconazole, terbinafine, and amphotericin B were defined as the lowest concentration of drug which resulted in total inhibition of fungal growth. For the assay, compound stock solutions were diluted two-fold with RPMI-1640 from 100 to 0.24 μg/mL (final volume = 100 μL) and a final DMSO (Sigma) concentration <1%. A volume of 100 μL of the inoculum suspension was added to each well with the exception of the sterility control where sterile water was added to the well instead. MIC was defined as the minimum inhibitory concentration of the compound, which resulted in total inhibition of fungal growth. Compounds with MICs ≥ 100 μg/mL were considered inactive.

## Figures and Tables

**Sch. 1.**
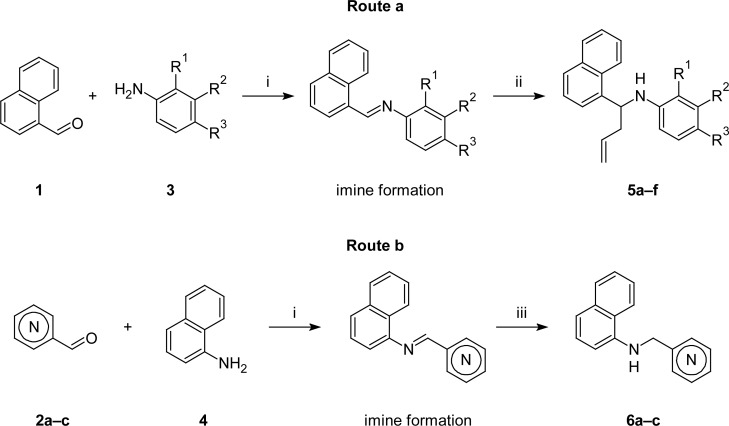
Synthetic routes to α-naphthalene-based compounds. Conditions and reagents: **i**: EtOH, reflux or CH_2_Cl_2_/Na_2_SO_4_/r.t.; **ii**: allylbromide/Mg/Et_2_O; **iii**: NaBH_4_/MeOH.

**Tab. 1.** Synthetized naphthylamines **5** and **6**

**Comp.**	**Type**	**Py ring**	**R^1^**	**R^2^**	**R^3^**	**Molecular formula**	**IR, *v*_NH_ (cm^−1^)**	**Physic. aspect**	**Yield (%)**
**5a**	A	–	H	H	MeO	C_21_H_21_NO	3403	red oil	80
**5b**	A	–	H	H	F	C_20_H_18_FN	3413	red oil	85
**5c**	A	–	H	H	Cl	C_20_H_18_ClN	3415	red oil	92
**5d**	A	–	H	H	Br	C_20_H_18_BrN	3413	yellow oil	94
**5e**	A	–	H	Cl	Cl	C_20_H_17_Cl_2_N	3415	red oil	81
**5f**	A	–	F	H	F	C_20_H_17_F_2_N	3425	red oil	98
**6a**	B	α	–	–	–	C_16_H_14_N_2_	3386	red oil	95
**6b**	B	β	–	–	–	C_16_H_14_N_2_	3355	red oil	91
**6c**	B	γ	–	–	–	C_16_H_14_N_2_	3299	yellow oil	94

**Tab. 2.** *In vitro* cytotoxic (IC_50_, μg/mL) and antifungal activities (MIC, μg/mL) of secondary naphthylamines **5** and **6**.

	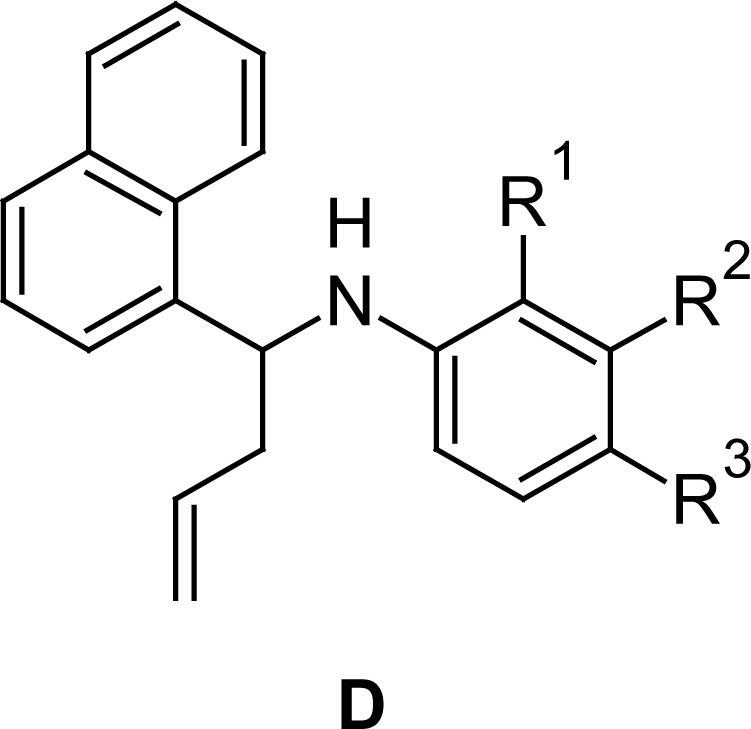			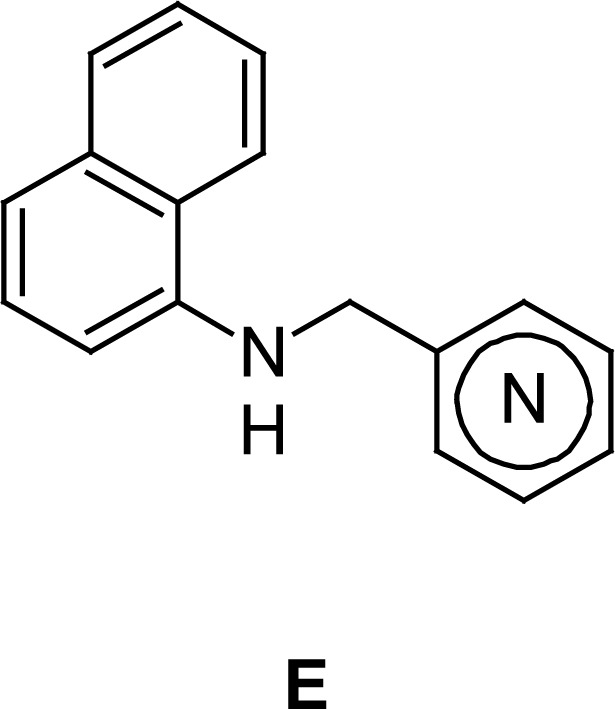		

**Cpd.**	Cytotoxicity*			Antifungal activity**		

	**IC_50_ (μg/mL)**			**MIC (μg/mL)**		

	**Cell-lines**			**Yeasts**	***Aspergillus***	**Dermatophytes**

	**MCF-7**	**H-460**	**SF-268**	***Ca, Ct, Sc, Cn***	***Afu, Afl, An***	***Mg, Tr, Tm***
**5a**	4.5 ± 0.5	4.8 ± 0.3	6.8 ± 0.4	i	i	i
**5b**	7.5 ± 0.3	8.9 ± 0.2	>10	i	i	i
**5c**	6.4 ± 0.5	7.7 ± 0.3	10.0 ± 0.4	i	i	i
**5d**	5.3 ± 0.5	5.9 ± 0.3	10.0 ± 0.4	i	i	i
**5e**	6.3 ± 0.5	8.9 ± 0.3	10.0 ± 0.4	i	i	i
**5f**	>10	>10	>10	i	i	i
**6a**	>10	>10	>10	i	i	i
**6b**	>10	>10	>10	50–62.5	50	6.25–25
**6c**	4.0 ± 0.5	3.3 ± 0.3	4.6±0.4	i	i	32–62
Adri	0.16 ± 0.1	0.18 ± 0.2	0.14 ± 0.1			
Amp				25–1.0	0.5–1.0	
Ket				0.12–0.5	0.12–0.5	
Terb						0.01–0.04

* Cytotoxic analysis was made in 96-well microtiter plates using the SRB assay. Cell lines used: breast MCF-7, lung H-460 and central nervous system SF-268 human cancer cell lines. Adri. = Adriamycin.

** Antifungal activity was determined with the microbroth dilution assay following the NCCLS guidelines. Fungi used: *C.a.: Candida albicans* ATCC10231, *C.t.: Candida tropicalis* C131; *C.n.:Cryptococcus neoformans* ATCC32264, *S.c.: Saccharomyces cerevisiae* ATCC9763, *A.n.: Aspergillus niger* ATCC9029, *A.fl.: Aspergillus flavus* ATCC 9170, *A.fu.: Aspergillus fumigatus* ATCC 26934, *M.g.: Microsporum gypseum* C 115, *T.r.: Trichophyton rubrum* C113, *T.m.: Trichophyton mentagrophytes* ATCC 9972. Amp. = Amphotericin B. Ket. = Ketoconazole. Terb. = Terbinafine. i = >100 μg/mL.

**Tab. 3.** Cytotoxic (IC_50_ in μg/mL) and antifungal activities (MIC in μg/mL) of **6a–c** tested from 100 to 0.01 μg/mL

	**Cytotoxicity**		

**Cpd.**	**IC_50_ (μg/mL)**		

	**MCF-7**	**H-460**	**SF-268**
**6a**	>10	>10	>10
**6b**	>10	>10	>10
**6c**	4.0 ± 0.5	3.3 ± 0.3	4.6 ± 0.4
Adri.	0.16 ± 0.1	0.18 ± 0.2	0.14 ± 0.1

	**Antifungal activity**									

**Cpd.**	**MIC (μg/mL)**									

	**Ca**	**Ct**	**Sc**	**Cn**	**Afu**	**Afl**	**An**	**Mg**	**Tr**	**Tm**
**6a**	>100	>100	>100	>100	>100	>100	>100	>100	>100	>100
**6b**	62.5	50	50	62.5	50	50	50	25	6.25	25
**6c**	>100	>100	>100	>100	>100	>100	>100	62	62	32
Amp.	1	0.5	0.5	0.25	0.5	0.5	0.5	–	–	–
Ket.	0.5	0.125	0.5	0.25	0.125	0.5	0.25	0.05	0.025	0.025
Terb.								0.04	0.01	0.04
